# An assessment of the potential miscalibration of cardiovascular disease risk predictions caused by a secular trend in cardiovascular disease in England

**DOI:** 10.1186/s12874-020-01173-x

**Published:** 2020-11-30

**Authors:** Alexander Pate, Tjeerd van Staa, Richard Emsley

**Affiliations:** 1 Division of Imaging, Informatics and Data Science, School of Health Sciences, Faculty of Biology, Medicine and Health, The University of Manchester, Oxford Road, Manchester, M13 9PL UK; 2 Department of Biostatistics and Health Informatics, Institute of Psychiatry, Psychology and Neuroscience, King’s College London, De Crispigny Park, London, SE5 8AF UK

**Keywords:** Cardiovascular disease risk prediction, Secular trend, Marginal structural model

## Abstract

**Background:**

A downwards secular trend in the incidence of cardiovascular disease (CVD) in England was identified through previous work and the literature. Risk prediction models for primary prevention of CVD do not model this secular trend, this could result in over prediction of risk for individuals in the present day. We evaluate the effects of modelling this secular trend, and also assess whether it is driven by an increase in statin use during follow up.

**Methods:**

We derived a cohort of patients (1998–2015) eligible for cardiovascular risk prediction from the Clinical Practice Research Datalink with linked hospitalisation and mortality records (*N* = 3,855,660). Patients were split into development and validation cohort based on their cohort entry date (before/after 2010). The calibration of a CVD risk prediction model developed in the development cohort was tested in the validation cohort. The calibration was also assessed after modelling the secular trend. Finally, the presence of the secular trend was evaluated under a marginal structural model framework, where the effect of statin treatment during follow up is adjusted for.

**Results:**

Substantial over prediction of risks in the validation cohort was found when not modelling the secular trend. This miscalibration could be minimised if one was to explicitly model the secular trend. The reduction in risk in the validation cohort when introducing the secular trend was 35.68 and 33.24% in the female and male cohorts respectively. Under the marginal structural model framework, the reductions were 33.31 and 32.67% respectively, indicating increasing statin use during follow up is not the only the cause of the secular trend.

**Conclusions:**

Inclusion of the secular trend into the model substantially changed the CVD risk predictions. Models that are being used in clinical practice in the UK do not model secular trend and may thus overestimate the risks, possibly leading to patients being treated unnecessarily. Wider discussion around the modelling of secular trends in a risk prediction framework is needed.

**Supplementary Information:**

The online version contains supplementary material available at 10.1186/s12874-020-01173-x.

## Background

Cardiovascular disease (CVD) risk prediction models such as QRISK are developed on longitudinal data spanning a long period of time (QRISK3 runs from 1998 to 2015 [1]). These models are updated each year to include the most recent data and at times remove old data. However, any secular trend in the outcome itself occurring within the time span of the development dataset is not modelled. Pate et al. [2] found a large downwards secular trend in CVD incidence over this time period in England. Downwards secular trends in the incidence of coronary heart disease, myocardial infarction, and stroke have also been reported in the literature [3–6]. Not including this trend in the prediction modelling could be resulting in the miscalibration of risk scores for patients in the present day, while including it would cause a large reduction in the predicted risks of these patients. Further research around this is needed, to quantify the impact of modelling this secular trend, and identify what is driving it and whether it should be modelled or not. In particular, it is important to clarify if the secular trend is being driven by an increase in statin use over time. In this scenario it should not be modelled, as it would result in risks predictions becoming lower and patients would be subsequently advised not to initiate statin treatment, despite this being the cause for the drop in risk.

In this paper we evaluate the effects of developing a model using the same methodology as QRISK3 (in the presence of the secular trend) and producing risk scores for patients in a time period after that of model development. We then propose an approach to incorporate secular trends in prediction models from longitudinal data, accounting for changes in treatment during follow up. This is formalised in four sequential analyses: A) quantifying the miscalibration in risk predictions of patients in the present day caused by this secular trend, B) assessing the sensitivity of the risk prediction model created to changes in patient characteristics, which could explain any miscalibration, C) an attempt to model the secular trend to remove miscalibration, D) developing a marginal structural model (MSM) to assess secular trend after adjusting for statin use during follow up.

## Methods

All analyses are carried out separately for male and female cohorts, as they have separate CVD risk prediction models in practice.

### Data source

A ‘CVD primary prevention cohort’ was defined from a Clinical Practice Research Datalink (CPRD) [7] dataset linked with Hospital Episode Statistics [8] (HES) and Office for National Statistics [9] (ONS) using the same criteria as QRISK3 [1]. The study period was 1st Jan 1998 to 31st Dec 2015 and the cohort entry date defined as the latest of: date turned 25; one year follow up as a permanently registered patient in CPRD; or 1st Jan 1998. Patients were excluded if they had a CVD event (identified through CPRD, HES or ONS) or statin prescription prior to their cohort entry date. The end of follow up was: the earliest date of patient’s transfer out of the practice or death; last data collection for practice; 31st Dec 2015 or five years follow up. Patients were censored after five years as five year risk predictions are used throughout this study. All predictor variables included in the QRISK3 [1] risk prediction model were extracted at cohort entry date. Code lists and detailed information on how variables were defined is provided in Additional file 1.

### Quantifying the miscalibration in risk predictions of patients in the present day

The first step was to quantify the miscalibration induced by developing a model over a time period in which a secular trend in CVD was present, and using it to calculate risk predictions for patients after this time period. Missing data for body mass index (BMI), systolic blood pressure (SBP), SBP variability, cholesterol, high density lipoprotein (HDL), smoking status and ethnicity in the CVD primary prevention cohort was imputed using multiple imputation by chained equations. The imputation model included all predictor variables from QRISK3, the Nelson Aalen estimation of the cumulative baseline hazard at the point of censoring or an event, and the outcome indicator. The package used to do this was mice [10]. Only one imputed dataset was produced, as running the analysis across multiple datasets and combining estimates was not essential to answering our hypotheses, and the computational time to do so was significant. Also the bespoke imputation procedure carried out on the data for developing the MSM (described later) resulted in a single dataset, so the decision was made across all analyses for consistency.

Patients were then split into two cohorts defined by their cohort entry date. Those with a cohort entry date prior to 1st Jan 2010 were put into the development cohort, with the remaining patients making up the validation cohort. Patients in the development cohort were then censored at 1st Jan 2010 if their follow up extended beyond this point. The data was split like this because if QRISK3 was replicated exactly using data from 1998 to 2015 for model development, it would not have been possible to assess the calibration of risk scores for patients after 2015, as they would have no follow up.

A Cox proportional hazards model using the same predictor variables as QRISK3 was then fit to the development cohort. Fractional polynomials of age, BMI and SBP were tested for using the mfp package [11]. Five year risk predictions were then generated for both the development and validation cohort using this model, and the calibration of these risks was assessed. Calibration was assessed by splitting individuals from the cohort into 10 groups by their predicted risk (deciles). The Kaplan Meier estimate of risk (observed risk) was then plot against the average predicted risk (predicted risk) within each decile. Eq. (1) corresponds to this model, where *h*(*t*) denotes the hazard function, *h*_0_(*t*) the baseline hazard at time *t*, *X*_0_ the vector of predictors at cohort entry date and *β*_*X*_ a vector of the associated coefficients .

1 $$ h(t)={h}_0(t)\ast \exp \left({\beta}_X.{X}_0\right) $$

### Attempt to model the secular trend to remove miscalibration in validation cohort

**Fig. 1:**
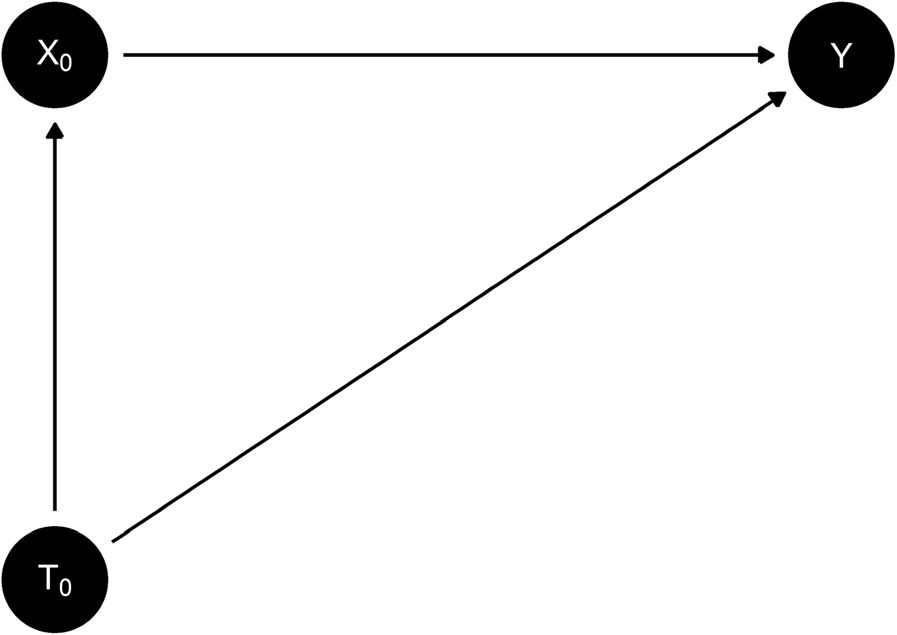
DAG-1

Given the miscalibration that was found in the validation dataset (see results), this indicated that the secular trend could not be explained by changes in predictor variables between the development and calibration dataset. This provided support for modelling the secular trend in the development cohort, to try and remove the miscalibration in the validation cohort. The same Cox model defined by eq. (1) was fitted to the development cohort, but with cohort entry date included as a variable, referred to as calendar time. This is denoted by *T*_0_ in Fig. 1 (DAG-1) and eq. (2). Unmeasured confounding is left off the DAGs to reduce the number of arrows and maintain clarity (particularly for DAG-2), however it may be present. The implications of unmeasured confounding are discussed in the limitations section (see exchangeability assumption).

All DAGs were generated using the dagitty software [12]. Fractional polynomials for this variable were tested using the mfp package [11]. Five year risks were generated for validation cohort and the calibration of the models was assessed.

2 $$ h(t)={h}_0(t)\ast \exp \left({\beta}_T.{T}_0+{\beta}_X.{X}_0\right) $$

### Developing an MSM to assess secular trend after adjusting for statin use during follow up

#### MSM – overview

A major concern was that an increase in statin use over time may have caused some of the reduction in CVD incidence. If the secular trend was driven by statin use, then modelling it (which would result in lower predicted risks) would make lots of patients whose risk if they remained untreated was > 10%, ineligible for treatment. Statin use at baseline could not have been driving this secular trend as the development cohort only considered patients who were statin free at baseline, however patients could initiate statins during follow up. The aim of this section was therefore to assess the presence of the secular trend when adjusting for statin use during follow up.

It is possible to adjust for changes in predictor variables and statin use post baseline using standard regression techniques (such as an interval censored Cox model). This would result in an estimate of the direct effect of calendar time on CVD incidence, the portion of which is not explained through changes in the predictor variables and statin use during follow up. Such a model would be sufficient for assessing whether the secular trend remained after adjusting for statin use during follow up in the development cohort. However the model could not be used in a risk prediction setting, as future values of predictor variables would be required to generate risk scores. When generating a risk score for a new individual, you would not know the future values of their predictor variables. Furthermore, the coefficient of statin use during follow up would not be causal, and the risk of a patient if they did/did not initiate statins during follow up could therefore not be estimated [13]. Therefore the proposed method to answer our question was an MSM.

**Fig. 2:**
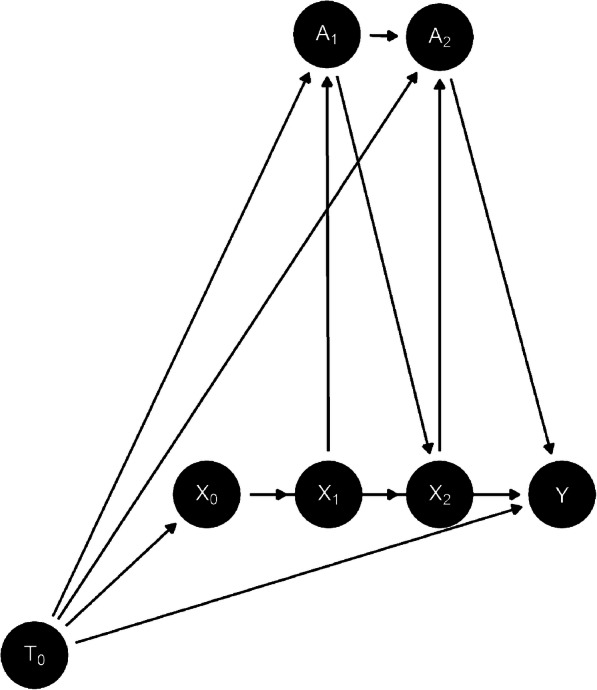
DAG-2

MSMs were developed to calculate the causal effect of a time dependent exposure on an outcome in an observational setting, where the treatment and outcome are confounded by time varying covariates [14, 15]. Sperrin et al. [13] have shown how MSMs can be used to adjust for ‘treatment drop in’, the issue of patients starting treatment during follow up in a dataset being used for risk prediction. Consider DAG-2 (Fig. 2), where *k* = 0 denotes baseline, and *k* = 1, 2 two time points during follow up (this could be extended to any number of time points). *A*_*k*_ denotes the statin treatment status at time *k*, *X*_*k*_ covariate information prior to time *k*, and *T*_*k*_ calendar time at time *k*. Note *A*_0_ is not included in DAG-2 as *A*_0_ = 0 by definition of the CVD primary prevention cohort. In the absence of unmeasured confounding, MSM’s allow for the estimation of $E\left[Y\left(\underset{\_}{A}=\underset{\_}{0}\right)|{X}_0\right]$, where A denotes the entire treatment course during follow up, as opposed to *E*[*Y*(*A*_0_ = 0)| *X*_0_]. The strategy involves adjusting for variables at baseline as normal and then re-weighting the population by variables that may be on the treatment causal pathway, breaking the links from *X*_*k*_ to *A*_*k*_. In the resulting pseudo population the allocation of treatment during follow up happens at random (within the levels of the variables defined at baseline). This allows the generation of risk scores using data at baseline only, but also accounting for statin use during follow up (the risk scores developed in a counterfactual scenario that no-one receives statin treatment). Importantly for this study, if calendar time only effected the outcome Y through increasing statin use in follow up, when using an MSM the direct effect of *T*_0_ on Y would be zero, and adjusting for calendar time at baseline would not result in a drop in the average risk score of patients in the validation cohort.

The estimator of $E\left[Y\left(\underset{\_}{A}=\underset{\_}{0}\right)|{X}_0\right]$ is only valid under the three identifiability assumptions of causal inference (exchangeability, consistency and positivity) and correct specification of the marginal structural model, and the model used to calculate the weights. The viability of these assumptions in this study is discussed in the limitations.

#### MSM - data derivation

The CVD primary prevention cohort was used as a starting point. However in order to derive the MSM, patient information was extracted at 10 time points, at 6 month intervals from the cohort entry date, denoted as *X*_*k*_ and *A*_*k*_ for *k* = 0, 1, 2,…, 9. The variable *X*_*k*_ contained all the QRISK3 predictors evaluated at time *k* (for test data this was the most recent value prior to time *k*). *A*_*k*_ = 1 if a patient had initiated statin treatment prior to *k*, and *A*_*k*_ = 0 otherwise. As patients were excluded from the cohort if they have had a statin prescription prior to their cohort entry date, A_0_ = 0 for all patients. If a CVD event happened within 6 months of a statin initiation, the statin initiation was ignored. This was to stop any effects of poorly recorded data (start of statins may have been triggered by the CVD event).

A key issue in deriving the dataset was missing data. A combination of imputation techniques were implemented to maintain consistency in variable information within each patient across the 10 time points. First, where possible, last observation carried forward imputation was implemented within each patient. Then, where possible, next observation carried backwards imputation was used to impute the remaining missing data. However, there was still missing data for patients who had no entries across all 10 time points for a given variable. The data at baseline was then extracted and missing values were imputed using one stochastic imputation. All predictor variables, Nelson Aalen estimate of baseline hazard and the outcome indicator were included in the imputation model (same process that was used to impute the data for the standard Cox model). These imputed baseline values were then used at each following time point (last observation carried forward imputation).

#### MSM - calculation of weights and specification of model

The MSM was fitted as a weighted interval censored Cox model using the coxph function from the survival package [16]. The weights themselves were calculated using the IPW package [17]. Stabilised weights were calculated as is common practice to provide more precise estimation of the weights. For individual *i*, the formula for the weight of interval/time period K was defined as:

3 $$ {sw}_i=\prod \limits_{k=0}^K{\left({\hat{p}}_{ki}^{\ast}\right)}^{A_{ki}}{\left(1-{\hat{p}}_{ki}^{\ast}\right)}^{1-{A}_{ki}}/\prod \limits_{k=0}^K{\left({\hat{p}}_{ki}\right)}^{A_{ki}}{\left(1-{\hat{p}}_{ki}\right)}^{1-{A}_{ki}} $$

where ${\hat{p}}_{ki}^{\ast }=P\left[{A}_k=1|{\underset{\_}{A}}_{k-1},{X}_0\right]$ and ${\hat{p}}_{ki}=P\left[{A}_k=1|{\underset{\_}{A}}_{k-1},{\underset{\_}{X}}_k,{X}_0\right]$, and ${\underset{\_}{A}}_k$ and ${\underset{\_}{X}}_k$ denote treatment history and covariate history respectively up time point *k* for individual *i*. More simply put, the denominator is the probability that the individual received the treatment they did, based on time varying predictors and predictors at baseline. The numerator is the probability that the individual received the treatment they did, based on predictors at baseline only. The models used to estimate the probability of treatment when deriving the weights were interval censored Cox models. If calendar time at baseline, *T*_0_, was being included in the MSM, it was also included as a stabilising factor in the calculation of the weights as part of *X*_0_. Detailed information on how to calculate weights is also given in the literature [15, 17, 18] and the formula for calculating weights (and notation for variables) matches that from the work by Sperrin et al. [13]

Two MSM’s were created, one that adjusted for calendar time at baseline and one that did not:

4 $$ h(t)={h}_0(t)\ast \exp \left({\beta}_A.{A}_t+{\beta}_X.{X}_0\right) $$

5 $$ h(t)={h}_0(t)\ast \exp \left({\beta}_A.{A}_t+{\beta}_X.{X}_0+{\beta}_T{T}_0\right) $$

The same fractional polynomials of age, BMI, SBP and calendar time that were found to be optimal in the standard Cox models were used in the MSM, and in the models used to calculate the weights. Ideally we would have re-calculated the optimal fractional polynomials for the weighted model fitted to the interval censored data, however software was not available to do this. Using the same fractional polynomials from the standard Cox analysis was preferred to having no fractional polynomials, as removing them led to poorly calibrated models. The coefficient *β*_*A*_ is the average causal effect of initiating statin treatment after adjusting for all other variables. It is quite common to allow the effect of statin treatment to be modified by baseline variables, which could be achieved by including interaction terms *A*_*t*_*X*_0_. However the primary aim was to account for statin use in follow up, rather than calculate the effect of statin treatment in different subgroups, so we did not feel this was necessary.

As a comparison, unweighted interval censored Cox models using only data at baseline (i.e. equation (1) and eq. (2) were fitted to the same data as the MSM. The effect of modelling the secular trend could then be assessed when using (interval censored) Cox regression, as well as under the MSM framework. This was preferred to re-using the standard Cox models directly, which were fitted to a different dataset.

#### MSM – analysis of interest

The MSM was used to generate risk predictions assuming no statin treatment at baseline or during follow up, $E\left[Y|{X}_0,\underset{\_}{A}=\underset{\_}{0}\right]$, the estimator of $E\left[Y\left(\underset{\_}{A}=\underset{\_}{0}\right)|{X}_0\right]$. The interval censored Cox model only produced risk predictions based on no statin treatment at baseline, *E*[*Y*| *X*_0_, *A*_0_ = 0], the estimator of *E*[*Y*(*A*_0_ = 0)| *X*_0_, ]. The outcome of interest was the risk ratio of the average predicted risk of patients in the validation cohort, before and after adjusting for calendar time at baseline in the MSM framework, $E\left[Y\left(\underset{\_}{A}=\underset{\_}{0}\right)|{X}_0,{T}_0\right]/E\left[Y\left(\underset{\_}{A}=\underset{\_}{0}\right)|{X}_0\right]$. This was compared to the risk ratio after adjusting for calendar time at baseline in the unweighted interval censored Cox models, (*E*[*Y*(*A*_0_ = 0)| *X*_0_, *T*_0_]/*E*[*Y*(*A*_0_ = 0)| *X*_0_]).

## Results

### Description of data

Differences between the development and validation cohorts are shown in Table 1. In the validation cohort, patients were generally younger and healthier (lower prevalence of comorbidities). The levels of missing data are reported in Table 2. The amount of missing data was lower in the validation cohorts compared to the development cohorts, and in the female cohorts compared to the male cohorts. The variables with highest levels of missing data (> 50% in some cases) were SBP variability, cholesterol/HDL ratio and ethnicity.

**Table 1:** Baseline variables in development and validation cohorts

	Male development	Male validation	Female development	Female validation
N	1,497,511	393,071	1,555,010	410,068
Age	43.07 (14.84)	37.18 (12.42)	44.56 (16.22)	37.4 (13.41)
BMI	26.07 (4.43)	26.3 (4.8)	25.54 (5.47)	25.78 (5.96)
Cholesterol/HDL ratio	4.51 (1.4)	4.32 (1.37)	3.76 (1.21)	3.52 (1.1)
SBP	130.67 (17.04)	127.71 (14.07)	125.15 (19.04)	119.53 (14.43)
SBP variability	10.37 (6.92)	9.39 (6.37)	9.66 (6.21)	8.87 (5.17)
Atrial fibrillation	0.61%	0.44%	0.48%	0.28%
Atypical anti-psychotic medication	0.25%	0.62%	0.23%	0.58%
Corticosteroid use	0.31%	0.22%	0.51%	0.36%
CKD stage 3/4/5	0.25%	0.57%	0.33%	0.95%
Diabetes (type 1)	0.26%	0.36%	0.19%	0.27%
Diabetes (type 2)	1.56%	0.93%	1.26%	0.78%
Ethnicity = Asian other	1.56%	2.84%	1.49%	2.88%
Bangladesh	0.34%	0.79%	0.24%	0.48%
Black	2.93%	5.80%	3.12%	5.90%
Chinese	0.45%	0.87%	0.56%	1.17%
Indian	2.49%	4.18%	2.21%	3.63%
Mixed	0.69%	1.47%	0.75%	1.64%
Other	1.53%	2.72%	1.45%	2.84%
Pakistan	0.92%	1.94%	0.76%	1.64%
White	89.09%	79.39%	89.42%	79.81%
Family history of CHD	10.67%	12.36%	14.89%	15.80%
HIV/AIDS	0.06%	0.19%	0.04%	0.13%
Migraine	2.71%	3.85%	6.73%	9.30%
Rheumatoid arthritis	0.28%	0.17%	0.74%	0.47%
Severe mental illness	4.59%	4.55%	9.07%	6.95%
SLE	0.01%	0.01%	0.09%	0.11%
Smoking = Never	47.37%	44.77%	57.03%	53.30%
Smoking = Ex	16.09%	20.59%	14.97%	22.49%
Smoking = Yes	36.53%	34.63%	28.00%	24.21%
Townsend = 1 (least deprived)	22.79%	17.30%	23.08%	17.70%
Townsend = 2	22.32%	18.38%	22.76%	19.03%
Townsend = 3	20.77%	20.82%	21.19%	21.17%
Townsend = 4	20.23%	22.85%	19.91%	22.53%
Townsend = 5	13.89%	20.65%	13.06%	19.57%
Treated hypertension	4.82%	3.28%	6.81%	3.81%

Mean (sd) is given of continuous variables, and proportions for categorical variables*. BMI* body mass index, *CKD* chronic kidney disease, *HDL* high-density lipoprotein, *SBP* systolic blood pressure, *SLE* systemic lupus erythematosus

**Table 2:** Amount of missing data in the development and validation cohorts

	Male Development	Male Validation	Female Development	Female Validation
SBP	41.49%	38.12%	20.18%	14.46%
SBP variability	80.18%	74.80%	51.92%	40.86%
BMI	49.56%	34.25%	34.36%	19.09%
Cholesterol/HDL ratio	59.13%	70.98%	56.59%	69.61%
Smoking	41.26%	10.32%	30.22%	4.37%
Ethnicity	69.42%	32.73%	65.60%	28.83%
Townsend	0.12%	0.07%	0.12%	0.06%

### Quantifying the miscalibration in risk predictions of patients in the present day

**Fig. 3:**
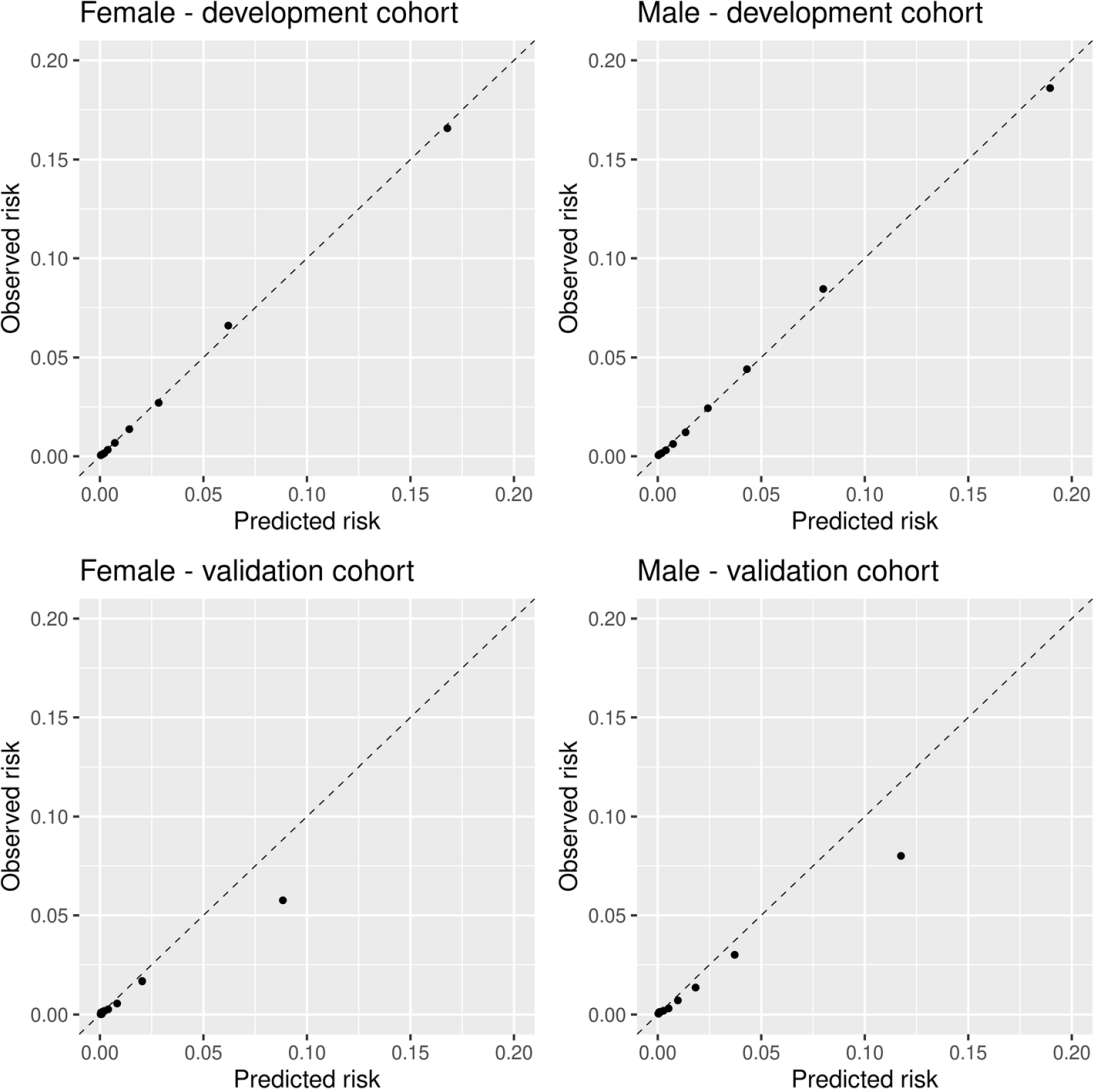
Model calibration in the development (pre 2010) and validation (post 2010) cohorts

Figure 3 shows the calibration of the model in the development and validation cohorts. While the model was well calibrated in the development cohort, as expected, there was a large under prediction of risks in the validation cohort. Statin prevalence and incidence rates in the primary prevention cohort are provided in Supplementary Tables 1 and 2 in Additional file 2.

### Attempt to model the secular trend to remove miscalibration in validation cohort

**Fig. 4:**
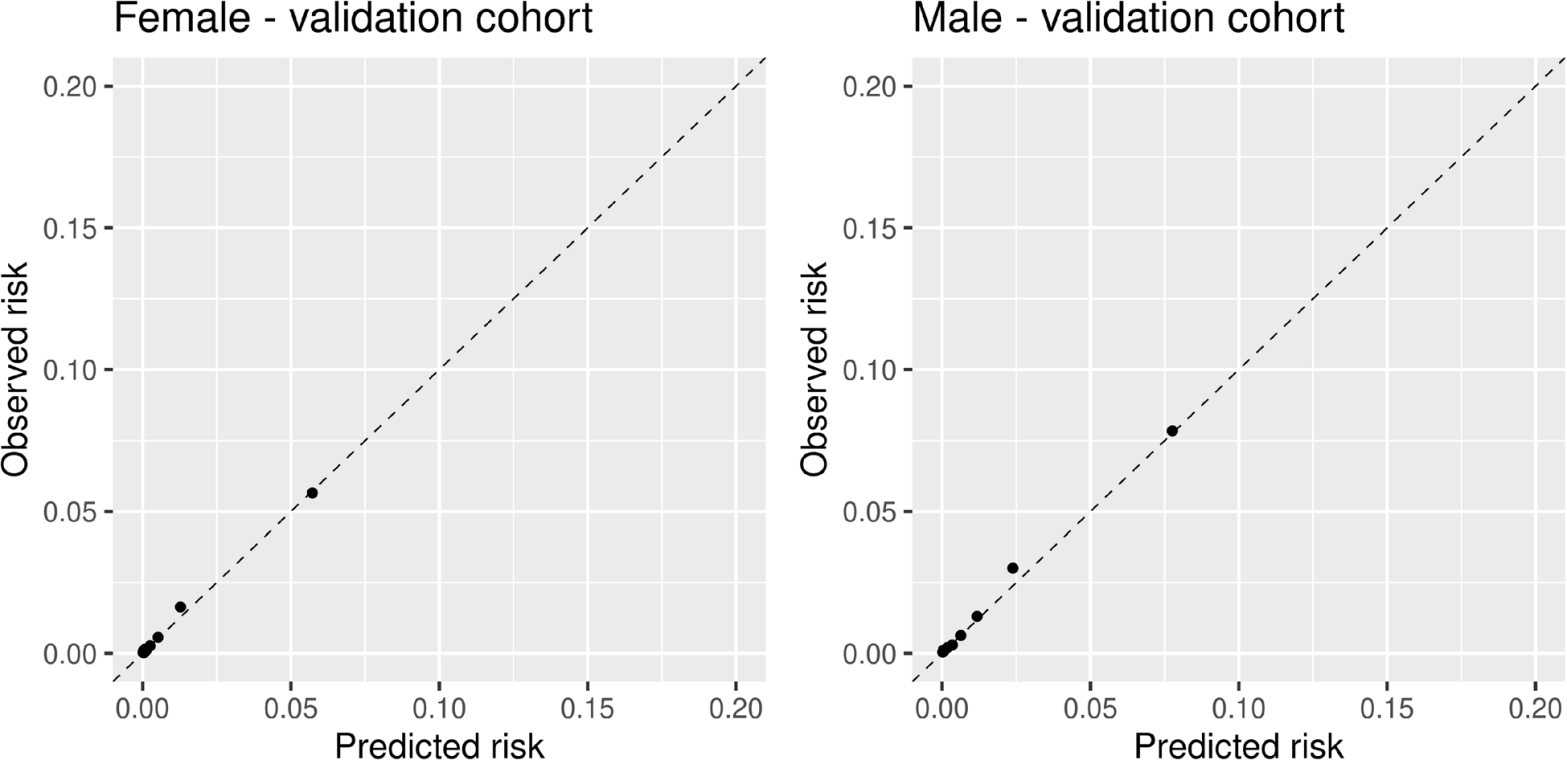
Calibration in the validation cohort when adjusting for calendar time

The calibration in the validation cohort after including secular trend into the model is shown in Fig. 4. There was still an under-prediction in the second highest risk group for both the female and male cohorts, but overall there was a substantive improvement in calibration compared to not modelling the secular trend.

### Developing an MSM to assess secular trend after adjusting for statin use during follow up

The average predicted risks of patients in the validation cohort before and after adjusting for calendar time, in the interval censored Cox and MSM setting, are presented in Table 3. The risk reduction caused by accounting for secular trend was marginally smaller under the MSM framework compared to the standard Cox. This means the effect of secular trend was slightly smaller when adjusting for statin use during follow up. However the difference would not be clinically significant, and there was still a large drop in risks. The hazard ratios from the two MSM’s are provided in Table 4, the coefficient of statin initiation is a causal estimate and can be used to help verify if the model has been derived correctly. Calibration of the interval censored Cox model and the MSM are presented in Supplementary Figs. 1 to 4 in Additional file 2, both are well calibrated.

**Table 3:** Average predicted CVD risk for patients in the validation cohort before and after secular trend was introduced, using an MSM and an interval censored Cox model

	Predicted CVD risk (average)		Relative reduction in risk
	Not adjusted for secular trend	Adjusted for secular trend	
Interval censored Cox			
Female	1.284%	0.826%	35.68%
Male	1.911%	1.274%	33.31%
Marginal structural model			
Female	1.287%	0.859%	33.24%
Male	1.941%	1.307%	32.67%

**Table 4:** Log hazard ratios (sd) of the categorical variables in the marginal structural model with and without secular trend included as a predictor variable

	Female		Male	
	Secular trend not accounted	Secular trend accounted	Secular trend not accounted	Secular trend accounted
Statin initiation	−0.34 (0.03)	− 0.26 (0.03)	− 0.29 (0.03)	− 0.22 (0.03)
Ethnicity: Asian other	− 0.05 (0.14)	0.07 (0.14)	− 0.01 (0.12)	0.10 (0.12)
Bangladeshi	0.24 (0.33)	0.35 (0.33)	0.71 (0.19)	0.80 (0.19)
Black	−0.11 (0.09)	−0.01 (0.09)	− 0.64 (0.10)	−0.56 (0.10)
Chinese	−0.22 (0.27)	−0.13 (0.27)	− 0.86 (0.30)	−0.77 (0.30)
Indian	0.24 (0.09)	0.31 (0.09)	0.20 (0.07)	0.26 (0.07)
Other ethnic group	−0.39 (0.16)	−0.31 (0.16)	− 0.19 (0.12)	−0.11 (0.12)
Pakistani	0.21 (0.18)	0.33 (0.18)	0.66 (0.11)	0.75 (0.11)
Townsend = 2	0.10 (0.02)	0.09 (0.02)	0.01 (0.01)	0.01 (0.01)
Townsend = 3	0.12 (0.02)	0.12 (0.02)	0.08 (0.01)	0.08 (0.01)
Townsend = 4	0.18 (0.02)	0.18 (0.02)	0.14 (0.01)	0.15 (0.01)
Townsend = 5 (most deprived)	0.31 (0.02)	0.30 (0.02)	0.24 (0.02)	0.23 (0.02)
Atrial fibrillation	0.68 (0.03)	0.68 (0.03)	0.53 (0.03)	0.53 (0.03)
Atypical antipsychotic medication	0.38 (0.07)	0.52 (0.07)	0.40 (0.09)	0.55 (0.09)
CKD stage 3/4/5	0.02 (0.05)	0.14 (0.05)	0.27 (0.05)	0.33 (0.05)
Corticosteroid use	0.49 (0.04)	0.49 (0.04)	0.44 (0.04)	0.42 (0.04)
Type 1 diabetes	0.84 (0.09)	0.84 (0.09)	0.41 (0.08)	0.40 (0.08)
Type 2 diabetes	0.65 (0.02)	0.63 (0.02)	0.60 (0.02)	0.58 (0.02)
Erectile dysfunction	NA	NA	0.16 (0.04)	0.23 (0.04)
Family history CHD	0.15 (0.01)	0.15 (0.01)	0.25 (0.01)	0.25 (0.01)
HIV	0.20 (0.71)	0.27 (0.71)	1.00 (0.19)	1.08 (0.19)
Hypertension	0.18 (0.01)	0.20 (0.01)	0.20 (0.01)	0.22 (0.01)
Migraine	0.17 (0.02)	0.17 (0.02)	0.19 (0.03)	0.19 (0.03)
Rheumatoid arthritis	0.28 (0.03)	0.28 (0.03)	0.25 (0.05)	0.25 (0.05)
Severe mental illness	0.36 (0.01)	0.33 (0.01)	0.28 (0.02)	0.26 (0.02)
Smoking = Ex	0.11 (0.01)	0.13 (0.01)	0.09 (0.01)	0.11 (0.01)
Smoking = Current	0.44 (0.01)	0.44 (0.01)	0.45 (0.01)	0.46 (0.01)
SLE	0.40 (0.13)	0.41 (0.13)	0.26 (0.28)	0.23 (0.28)

*CHD *coronary heart disease*, CKD* chronic kidney disease, *SLE* systemic lupus erythematosus

## Discussion

This results in this paper show that not modelling the secular trend in CVD incidence in England causes over prediction of risks for patients in the present day. Also, the secular trend in CVD incidence cannot be explained by changes in statin use over time, because when adjusting for calendar time in the MSM framework the risk predictions of patients in the validation cohort still dropped substantially.

These findings support the need to adjust for calendar time in prediction models used to drive clinical decision making in England. However the drop in risk caused by accounting for this secular trend is drastic and changes should not be made in practice without the generation of more evidence. Most importantly, these findings should be reproduced in a different dataset. This should not be difficult as QRISK3 has been developed in the QResearch database, and QRISK2 has been externally validated in the Health Improvement Network database [19]. This means analysis ready datasets exist and could be tested for secular trends in CVD with minimal extra work.

The next step would then be to try and identify what is causing this drop in CVD incidence. In this study, we ruled out one potential cause, the use of statins during follow up. The secular trend could also be driven by increasing use of other CVD medications, such as antihypertensives. We focused on statins as this is the recommended treatment for primary prevention of CVD, but the impact of other medications should also be explored. This could be done on a simple level, by assessing how many patients in the development cohort initiate the medications of interest during follow up. If the number of individuals doing so looks to be significant, then the impact could be assessed formally using the same techniques as this study. Another possible cause of the secular trend could be changes in recording practices. If this was the true cause this would be another reason not to model the secular trend, as it would not represent a true change in the underlying disease process. Primary care records in particular may be susceptible to differential recording over time as monetary incentives are given for recording specific things. However, a large portion of the events are identified in HES and ONS which will not have suffered from the same level of differential recording. This is backed up by the trends reported in the literature, which are also not based on primary care codes [3–6]. Further work in a causal framework to establish what is causing this drop would be really valuable and could provide a much stronger argument for modelling the secular trend (e.g. if its driven by lifestyle changes). However, given the current evidence, there is still not a strong argument against modelling it.

Risk scores should be based on current data; this is why the series of QRISK models have used a rolling window for their development datasets. If there was a much higher incidence of CVD in the 1990s due to various differences in healthcare management, we would not want to incorporate this into current risk scores as it would inflate the risks. Therefore, there is also no reason to assume the incidence of CVD has been the same throughout the time window of data we are using. In this sense, current approaches to risk prediction are contradictory. We are happy to omit old data from our cohort periodically to reflect changes in the population; but we are not willing to model changes in the population over the time period in which we have defined our cohort. If wanting to do so, dynamic models are what should be used to model changes over time.

With respect to the dynamic modelling methods outlined by Jenkins et al., [20] the current approach in England implemented by QRISK series is discrete model updating (models are re-calculated in a more recent dataset each year). In this study we modelled the secular trend by including a calendar time variable at baseline. This effectively allowed the intercept (or baseline hazard) to vary by calendar time, and is a special case of a varying coefficient model. However, there are more complex methods such as Bayesian model updating and varying coefficient models that allow changes in predictor coefficients over time, and could give more control over how the secular trend is modelled. If a dynamic model was to be developed for use in practice, these methods should be considered, alongside how to use these methods within an MSM framework. Arguably the use of an MSM should be standard procedure in the presence of ‘treatment drop in’ during follow up, as a normal Cox model under predicts the risk of patients if they were to remain untreated, which is what treatment decisions should be based on [13]. If modelling a secular trend in the outcome that was being partially driven by this treatment drop in (which was not the case in this study), it would be even more important to work under an MSM framework. However, currently it is not clear how the more complex dynamic modelling approaches would be handled in an MSM framework. This is therefore a key area for future research.

It is worth noting that it may not be necessary to build an MSM in order to rule out treatment initiation as the cause for a secular trend. The method was used in this paper to highlight the potential for MSM’s to be used in this manner. In practice, it would be sensible to look at the overall rate of treatment initiation (and prevalence) first. The incidence and prevalence rates of statin treatment in this cohort are provided in Additional file 2. We see that statin initiation rates range between 10 and 20 initiations per 1000 person years (which works out at about 1–2% of the population each year). Given the hazard ratios of statin initiation (between 0.7–0.8), even if everybody stopped statin initiation, this would affect too small of a proportion of the cohort to be driving the drop in risk. Therefore in this particular scenario, the use of an MSM may not have been required to rule out statins as the cause for the secular trend. There are however strong arguments for MSM’s to be used in practice regardless of the presence of secular trends, in order to appropriately estimate patient risk if they were/were not to initiate treatment.

### Limitations

There are several limitations to the study. The first is that the estimate of $E\left[Y\left(\underset{\_}{A}=\underset{\_}{0}\right)|{X}_0\right]$ is only valid if the assumptions of exchangeability, consistency, positivity (identifiability assumptions) and correct model specification are all met. The untestable assumption of exchangeability, or no unmeasured confounding, represents the fundamental problem with deriving causal estimates from observational data. Specifically with regards to our model, this is violated if there are any other variables which predict both treatment exposure and the outcome of CVD. With respect to DAG-2, this would be shown by another node with arrows going into both *A*_*k*_ and the outcome Y. If violated the estimate of statin treatment will be biased (and subsequently the risk scores conditional on no statin treatment during follow up will be biased too). Given the large number of predictors available we hope that the unmeasured confounding is not too extensive. The consistency assumption, that a subject’s counterfactual outcome under their observed exposure history is precisely their observed outcome, is generally considered a reasonable assumption when estimating the effects of medical treatments [18]. This is maybe less true in our data as a patient could initiate statins any time over a 6 month period and be assigned the same exposure value. However we did not believe that initiating within a 6 month interval would have a significant impact on the outcome, and reducing the size of the intervals would have been impractical. The positivity assumption, that there were unexposed and exposed individuals at every level of the confounders, was reasonable given the large size of the development dataset and the resulting number of statin initiations.

The assumption of correct model specification, as is the case with all models, will have been violated to some extent in this study. For example, the fractional polynomials of continuous variables calculated from the standard Cox models were used in the MSM. It was not clear how to estimate optimal functional forms under the MSM framework, but re-using the functional forms from the Cox models provided better model performance than just having linear terms. Also, not all variables and interaction terms from the MSM were used in the model to calculate the weights. Doing so produced extreme values weights, and therefore variables in the weighting models were chosen to minimise this. This follows the advice of Cole and Hernan, who state “*one may wish to omit control for weak confounders that cause severe non-positivity bias because of a strong association with exposure*” [18]. There is no clear-cut way to do this, and therefore a more appropriate set of predictors in the weighting model may have existed. Finally, we only considered the effect of initiating statin treatment. A more detailed MSM which also modelled discontinuation from treatment would allow the calculation of a patients risk if they were to initiate treatment at baseline and not discontinue (or discontinue after a fixed period of time), as opposed to just the risk if they initiate treatment at baseline. However, the density of data available in CPRD, or any other primary care electronic health record is probably not sufficient for this. To model statin initiation and discontinuation at that granularity, more regular updates on predictor variables would be required.

The second limitation was that the results are not directly applicable to the models used in practice in the UK, which are based on 10-year risk scores. However, we have no reason to think the results would not be generalizable because a similar secular trend was found in previous work when dealing with 10-year risks [2]. The third limitation was the level of missing data. Changes in the time varying predictor variables is what drives the weighting in the MSM in order to calculate the effect of statin initiation. Therefore not having predictor information at each time point, and re-using predictor information from previous time points may have led to a biased estimate of statin initiation.

One way to assess the potential impact of limitations 1 (violating assumptions) and 3 (missing data) was to check the hazard ratio for initiating statin treatment (ranging between 0.71–0.81) was in a sensible range. We compared this to the effect estimates of statins from trials reported in the appendices of the NICE guidelines (see section L.2.3.4), [21] and there is reasonable agreement. It should be noted that they report relative rates for specific CVD outcomes which are not directly comparable to our composite definition. However, the similarities that exist still ease concerns over limitations 1 and 3, and that the model was well specified despite these limitations.

## Conclusions

In conclusion, inclusion of the secular trend into the model substantially changed the CVD risk predictions. Models that are being used in clinical practice in the UK do not model secular trend and may thus overestimate the risks, possibly leading to patients being treated unnecessarily.

## Supplementary Information

**Additional file 1.** Predictor variables and code lists. A breakdown of predictor variables and how they were derived, and full code lists used to extract data from the raw electronic health record.

**Additional file 2.** Supplementary tables and figures that are referenced in the main manuscript.

## Data Availability

The datasets generated and/or analysed during the current study are not publicly available as this would be a breach of the contract with CPRD. However it can be obtained by a separate application to CPRD after getting approval from Independent Scientific Advisory Committee (ISAC). To apply for data follow the instructions here: https://www.cprd.com/research-applications . The code used for running analyses is provided at the following GitHub page: https://github.com/alexpate30/An-assessment-of-the-potental-miscalibration

## Abbreviations

BMI: Body mass index
CPRD: Clinical Practice Research Datalink
CKD: Chronic kidney disease
CVD: Cardiovascular disease
DAG: Directed Acyclic Graph
HES: Hospital episode statistics
HDL: High-density lipoprotein
ONS: Office for National Statistics
MSM: Marginal Structural Model
SBP: Systolic blood pressure
