# Supplementary material for: An assessment of the potential miscalibration of cardiovascular disease risk predictions caused by a secular trend in cardiovascular disease in England
Source: BMC Med Res Methodol. 2020 Nov 30;20:289. doi: 10.1186/s12874-020-01173-x (PMC7706224; doi:10.1186/s12874-020-01173-x)

# Supplementary tables and figures

## Prevalence of statin use and incidence of CVD

Supplementary Table 1: Prevalence of statin use each year in CVD primary prevention cohort

| Year | Total follow up in years | Prescriptions | Prescriptions per 1000 person years |
| --- | --- | --- | --- |
| 1998 | 738031 | 8585 | 11.63 |
| 1999 | 837959 | 27044 | 32.27 |
| 2000 | 1040735 | 56827 | 54.60 |
| 2001 | 1295439 | 106348 | 82.09 |
| 2002 | 1429880 | 186787 | 130.63 |
| 2003 | 1591167 | 310417 | 195.09 |
| 2004 | 1648158 | 488761 | 296.55 |
| 2005 | 1700595 | 661788 | 389.15 |
| 2006 | 1744492 | 870761 | 499.15 |
| 2007 | 1775518 | 1030767 | 580.54 |
| 2008 | 1801027 | 1142698 | 634.47 |
| 2009 | 1799115 | 1240413 | 689.46 |
| 2010 | 1777610 | 1263314 | 710.68 |
| 2011 | 1712269 | 1217875 | 711.26 |
| 2012 | 1669433 | 1216451 | 728.66 |
| 2013 | 1546937 | 1152425 | 744.97 |
| 2014 | 1325230 | 1000030 | 754.61 |
| 2015 | 1051748 | 806999 | 767.29 |
| 2016 | 204290 | 154993 | 758.69 |
| Total | 26689633 | 12943283 | 484.96 |

Supplementary Table 2: Rate of initiation of statin treatment each year in CVD primary prevention cohort

| Year | Total follow up in years | Number initiated | Incidence rate per 1000 person years |
| --- | --- | --- | --- |
| 1998 | 738031 | 2133 | 2.89 |
| 1999 | 837959 | 3391 | 4.05 |
| 2000 | 1040735 | 5664 | 5.44 |
| 2001 | 1295439 | 9587 | 7.40 |
| 2002 | 1429880 | 15370 | 10.75 |
| 2003 | 1591167 | 22554 | 14.17 |
| 2004 | 1648158 | 31251 | 18.96 |
| 2005 | 1700595 | 30791 | 18.11 |
| 2006 | 1744492 | 37520 | 21.51 |
| 2007 | 1775518 | 29573 | 16.66 |
| 2008 | 1801027 | 29384 | 16.32 |
| 2009 | 1799115 | 28322 | 15.74 |
| 2010 | 1777610 | 22398 | 12.60 |
| 2011 | 1712269 | 18499 | 10.80 |
| 2012 | 1669433 | 19326 | 11.58 |
| 2013 | 1546937 | 16623 | 10.75 |
| 2014 | 1325230 | 13255 | 10.00 |
| 2015 | 1051748 | 10295 | 9.79 |
| 2016 | 204290 | 2330 | 11.41 |
| Total | 26689633 | 348266 | 13.05 |

## Calibration of the marginal structural model and interval censored Cox model

Calibration of the marginal structural models and interval censored Cox models (used to calculate reduction in risk in Table 3) are presented here. When assessing the calibration of the MSM (in either the development or validation cohorts), it was done on the subset of patients who received no statin treatment during follow up. This is because the risk scores generated were conditional on having no statin treatment during follow up. The calibration of the interval censored Cox model was carried out on the entire development/validation cohorts. The interval censored Cox model would under predict the risk of patients who do not receive statins during follow up.

Supplementary Figures 1 and 2 show that when secular trend was not adjusted for under the MSM setting, there was a significant under prediction of risks in the validation cohort, which could be accounted for by modelling calendar time. This is very similar to the non-MSM setting, for which the calibration of the interval censored Cox model is presented in Supplementary Figures 3 and 4.

Supplementary Figure 1: Calibration of the MSM in the validation cohort, with or without adjustment for calendar time (male cohort)


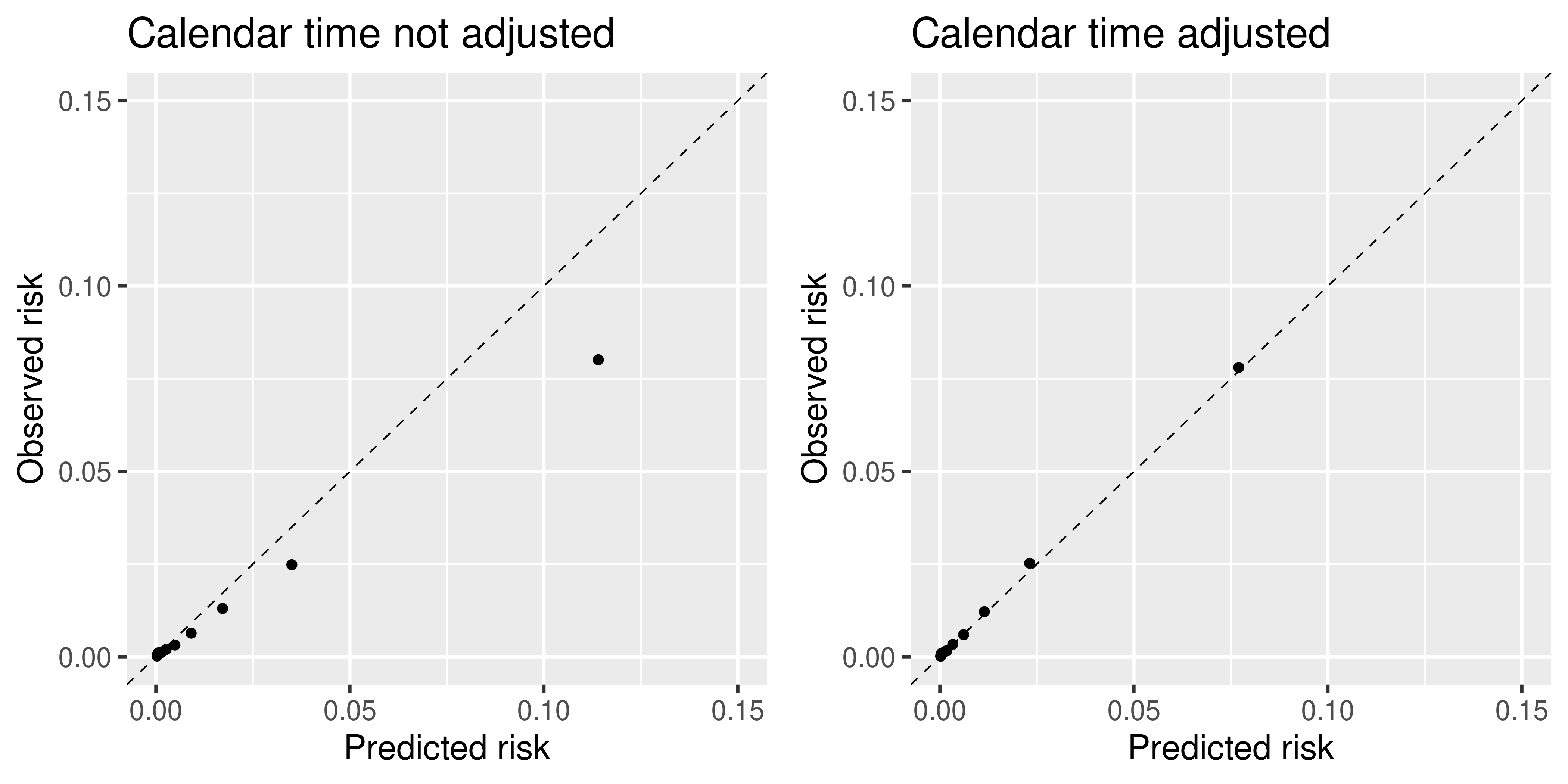


Supplementary Figure 2: Calibration of the MSM in the validation cohort, with or without adjustment for calendar time (female cohort)


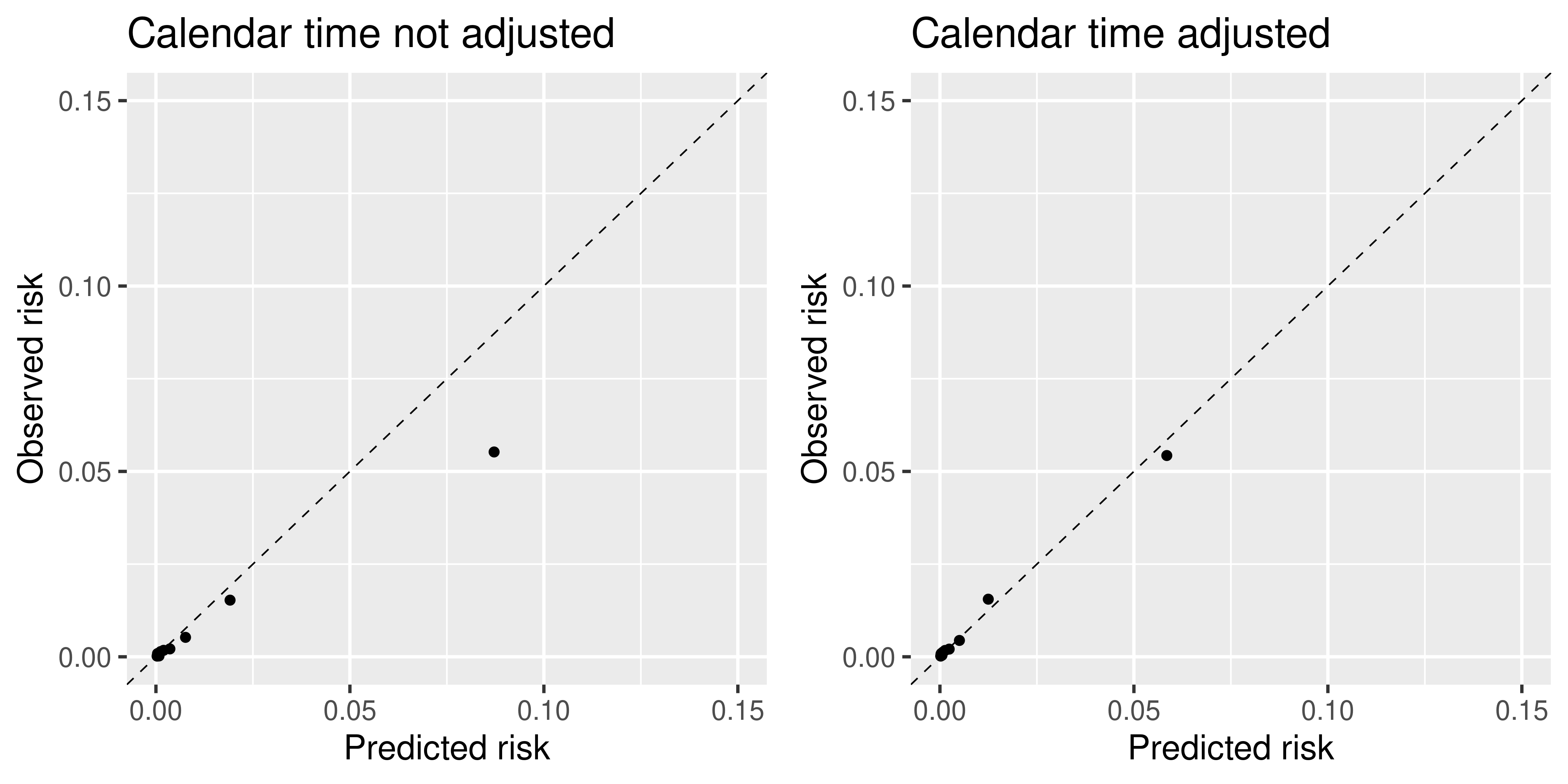


Supplementary Figure 3: Calibration of the interval censored Cox model in the validation cohort, with or without adjustment for calendar time (male cohort)


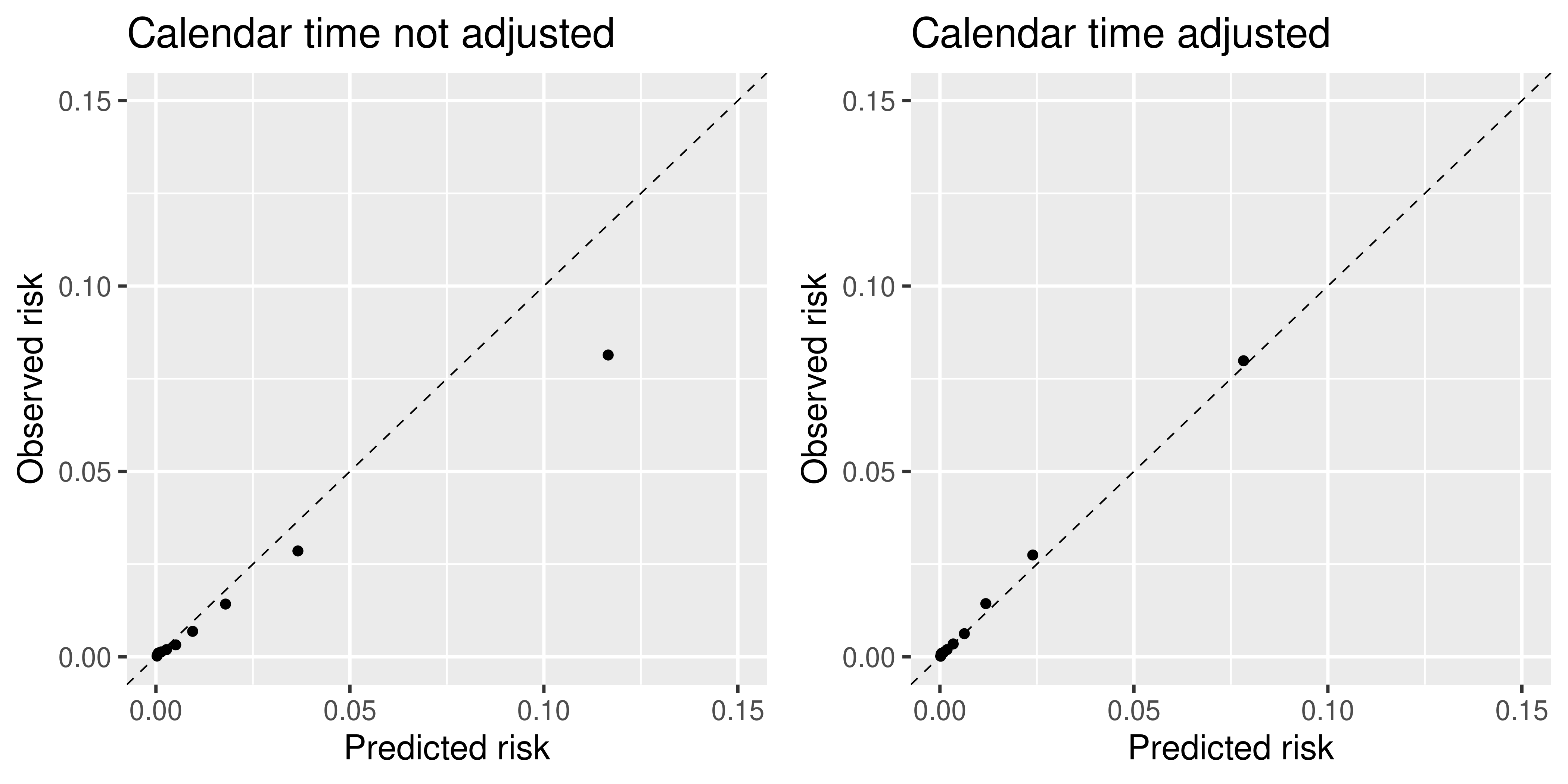


Supplementary Figure 4: Calibration of the interval censored Cox model in the validation cohort, with or without adjustment for calendar time (female cohort)


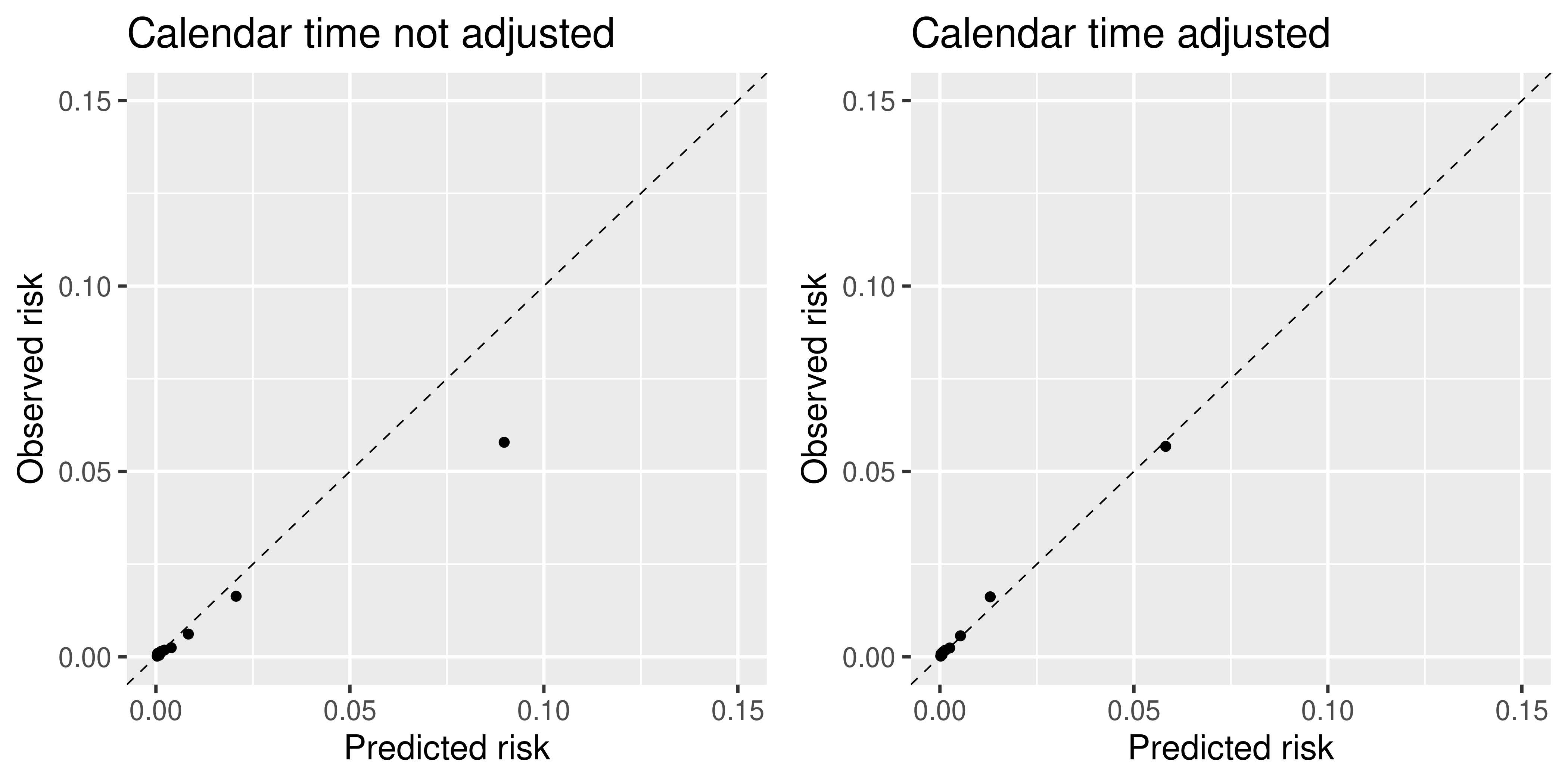


It is worth noting here that after adjusting for calendar time, the calibration is slightly poorer than in section 5.4.3, where standard cox models were used on data at baseline. We hypothesise this is because we were unable to test for fractional polynomials of the continuous variables when running an interval censored Cox model, meaning we re-used the same fractional polynomials from the standard Cox regression carried out in section 5.4.3. Given that the data was imputed differently, these may not be the best fractional polynomials to use. We did compare them to using no fractional polynomials, and the models were better calibrated when including them.

## Comparison of predicted risks in development and validation cohort

We wanted to check that the model was in fact reacting to the changes at baseline beween the development and validation cohort. There is a significant change in the characteristics of the cohort. This should result in a drop in risk in the validation cohort compared to the development cohort. This is not an exact test with a clear outcome, and the results were interpreted by the authors.

As shown in Supplementary Figure 5, the predicted risks in the validation cohort were significantly smaller than those in the development cohort. This indicates that the model did appropriately reflect the differences in baseline predictors between the cohorts, and the secular trend in CVD incidence could not be explained by this.

***Supplementary Figure 5:*** *Comparisons of predicted risks in development and validation cohorts*


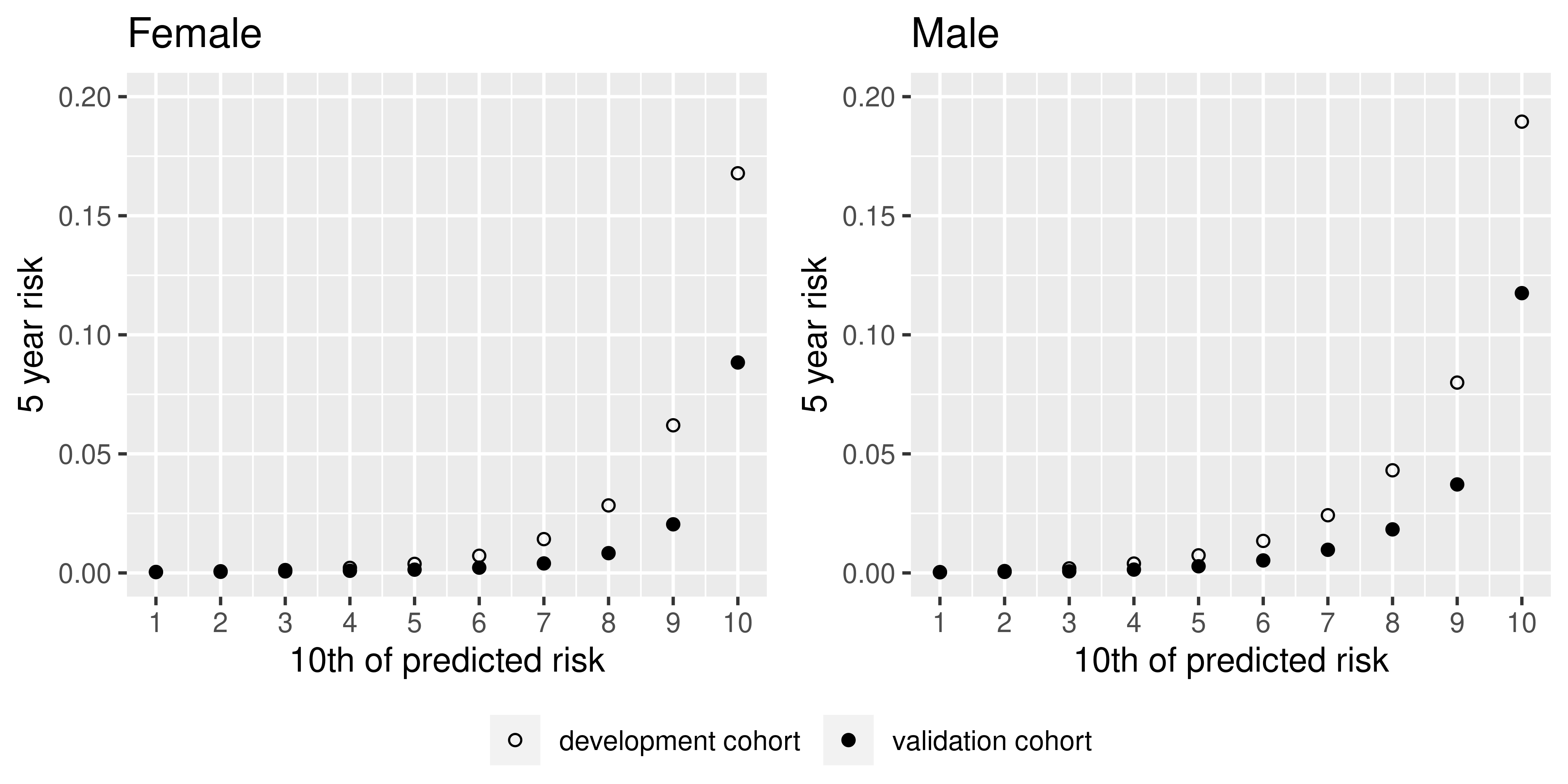

Supplement: Supplementary file 2 — Additional file 2. Supplementary tables and figures that are referenced in the main manuscript. [file 12874_2020_1173_MOESM2_ESM.docx]
